# Small molecular weight alginate gel porogen for the 3D bioprinting of microvasculature

**DOI:** 10.3389/fbioe.2024.1452477

**Published:** 2024-09-24

**Authors:** Florian Vanlauwe, Charlotte Dermaux, Sabina Shamieva, Stef Vermeiren, Sandra Van Vlierberghe, Phillip Blondeel

**Affiliations:** ^1^ Tissue Regeneration and Organ Printing (TROP) Research Center, Department of Plastic and Reconstructive Surgery, Ghent University Hospital, Ghent, Belgium; ^2^ Polymer Chemistry and Biomaterials Group, Centre of Macromolecular Chemistry (CMaC), Department of Organic and Macromolecular Chemistry, Ghent University, Ghent, Belgium

**Keywords:** extrusion bioprinting, 3D microvascularization, porogen, angiogenesis, vasculogenesis, spheroid bioprinting, gelatin methacryloyl bioink, scaffold

## Abstract

In order to recreate the complexity of human organs, the field of tissue engineering and regenerative medicine has been focusing on methods to build organs from the bottom up by assembling distinct small functional units consisting of a biomaterial and cells. This bottom-up engineering requires bioinks that can be assembled by 3D bioprinting and that permit fast vascularization of the construct to ensure survival of embedded cells. To this end, a small molecular weight alginate (SMWA) gel porogen is presented herein. Alginate is a biocompatible biomaterial, which can be easily converted into small porogen gels with the procedure reported in this article. The SMWA porogen is mixed with photo-crosslinkable hydrogels and leached from the hydrogel post-crosslinking to increase porosity and facilitate vascularization. As a proof of concept, this system is tested with the commonly used biomaterial Gelatin Methacryloyl (GelMA). The SMWA porogen-GelMA blend is proven to be bioprintable. Incubating the blend for 20 min in a low concentration phosphate buffered saline and sodium citrate solution significantly reduces the remaining porogen in the hydrogel . The intent to completely leach the porogen from the hydrogel was abandoned, as longer incubation times and higher concentrations of phosphate and citrate were detrimental to endothelial proliferation. Nonetheless, even with remnants of the porogen left in the hydrogel, the created porosity significantly improves viability, growth factor signaling, vasculogenesis, and angiogenesis in 3D bioprinted structures. This article concludes that the usage of the SMWA porogen can improve the assembly of microvasculature in 3D bioprinted structures. This technology can benefit the bottom-up assembly of large scaffolds with high cell density through 3D bioprinting by improving cell viability and allowing faster vascularization.

## 1 Introduction

In the last 40 years, tissue engineering and regenerative medicine have made huge progress towards the biofabrication of three-dimensional (3D) tissue analogues for use in drug screening, disease models, and organ transplants (Gaglio et al., 2024; Olson et al., 2011). Biofabrication methods in tissue engineering and regenerative medicine are generally subdivided into two main groups: top-down and bottom-up. In the top-down approach, first a 3D scaffold is fabricated, which simulates the ECM and the general shape of the organ, followed by seeding the cells onto this scaffold (Caddeo et al., 2017). However, this method lacks the ability to recreate the complex design of human organs, which entails a combination of different cell types with different functions in an intricate 3D architecture (Nichol and Khademhosseini, 2009). Conversely, in bottom-up tissue engineering, the 3D tissue is fabricated by combining small functional units, containing specific cells and/or biomaterials, via a modular assembly approach. These functional units can be primed for development towards executing a certain tissue function before assembly into an organ, which permits the recreation of the physiological complexity of human tissues/organs (Elbert, 2011).

To assemble these functional units in 3D, technologies such as 3D printing have been introduced. In this regard, extrusion-based bioprinting (EBB) is the most thoroughly documented method because of its affordability and ease of use. Towards the fabrication of human-sized organs, this type of bioprinting is preferred as it better supports the printing of larger constructs with high aspect ratio and cell densities (Datta et al., 2017; Murphy and Atala, 2014; Wüst et al., 2011). Moreover, EBB is compatible with multiple printing heads, which allow the deposition of multiple materials and cell types in a single construct. This multimaterial deposition system is essential to assemble the functional units in bottom-up tissue engineering (Nguyen et al., 2023). In the context of EBB and the construction of large, well-defined structures, the bioink (comprising biomaterials and cells) must meet two main requirements in addition to being biocompatible: First, the biomaterial needs to have favorable rheological features, such as shear thinning properties, to be compatible with EBB. The shear-thinning effect describes a reversible decrease in the viscosity of a bioink as the shear rate increases. This allows the facile and cell compatible extrusion through the nozzle under pressure and permits the biomaterial to structurally recover and retain its shape post-deposition (Liu et al., 2017; Barrulas and Corvo, 2023). Secondly, as EBB has insufficient resolution to create capillary-sized structures, the bioink needs to permit the development of a capillary system through self-assembly of the endothelial cells (ECs) either through cultivation *in vitro* or by sprouting angiogenesis from a host organism after implantation *in vivo* (Barrs et al., 2020). A vascular network is necessary, as diffusional limits for oxygen and nutrients inhibit the survival of tissues, thicker than about 200 μm and with a cell density exceeding 3 × 10^5^ cells/cm^3^ (Fleischer et al., 2020).

*In vitro* fabrication of a self-assembling capillary system was first described by Kubota et al., in 1988. They observed that ECs, cultured on a 2D Matrigel, were able to form vessel-like networks (Kubota et al., 1988). These vessel-like networks were shown to be capable of anastomosing and being perfused by a host organism (Levenberg et al., 2002). However, the vessels formed solely by ECs proved to be highly unstable. A more stable formation of vessels was achieved by co-culturing ECs with mesenchymal cell types, which fulfilled a pericyte-like function (Chen et al., 2012; Jain, 2003). In the same way, tissue spheroids can establish a vascular network by combining ECs with mesenchymal stem cells during spheroid formation in a non-adhesive environment (Benmeridja et al., 2020).

Hydrogels constituting natural polymers have been extensively used for microvascular tissue engineering (Blache and Ehrbar, 2018). Gelatin, which originates from the denaturation of collagen, is a popular natural biomaterial because of its low cost and biocompatibility (Bello et al., 2020). It contains RGD-amino acid sequences for cell attachment and features the shear thinning effect needed for extrusion bioprinting (Kokol et al., 2021). Gelatin solubilizes above 30°C, and thus, needs an additional modification to maintain its 3D structure at the cell culturing temperature of 37°C. One such enhancement is the addition of methacryloyl groups to gelatin to form gelatin methacryloyl (GelMA), which allows biocompatible chemical crosslinking of the gelatin proteins in the presence of a photo-initiator and UV light (Billiet et al., 2014; Van Den Bulcke et al., 2000). To date, GelMA is one of the most widely used bioinks for extrusion bioprinting (Shi et al., 2023).

To improve vascularization efforts in hydrogel systems, research groups have tried to increase porosity in bioprinted hydrogel structures. A first method to increase porosity is by bioprinting the pore structures directly (De Moor et al., 2021). However, to maximize migration and cell signaling, the pores need to be interconnected in the hydrogel and reach all the embedded cells directly to prevent trapping of the cells inside the hydrogel (Han et al., 2013). Bioprinting a pore system that fulfills these requirements is extremely difficult due to the lacking resolution of EBB (Ouyang et al., 2022; Somo et al., 2015). To circumvent this, an extensive pore system can be established after 3D bioprinting the hydrogel construct. In this regard, cryogelation and lyophilization of printed hydrogels have been described. However, these methods are not biocompatible towards cells embedded in the biomaterial (Bao et al., 2020). Alternatively, a pore-creating agent, called “porogen”, can be mixed with a cell-laden bioink, bioprinted, and removed after printing to create the pores in the hydrogel structure (Han et al., 2013). In the present work, we explore calcium-crosslinked alginate as a porogen to increase porosity of the hydrogel, thereby improving vascularization within a 3D bioprinted construct.

## 2 Materials and methods

### 2.1 GelMA synthesis

GelMA was prepared according to the protocol described by Van Den Bulcke et al. (2000). Briefly, 100 g of gelatin was dissolved in 1 L of phosphate buffer (pH 7.8) at 40°C with continuous stirring. Once the gelatin was fully dissolved, 2.5 equivalents (96.25 mmol) methacrylic anhydride (Sigma-Aldrich, 276685), with respect to the primary amines present in (hydroxyl) lysine and ornithine (38.5 mmol primary amines/100 g), were added. This mixture was then stirred vigorously for 1 h to ensure thorough mixing and reaction. After the reaction was completed, 1 L of double-distilled water (DDW) (Milli-Q) water was added to the mixture. The solution was then dialyzed in membranes with a molecular weight cut-off (MWCO) of 12k-14k Dalton (Da) (Spectra/Por^®^ 4, 132706) in distilled water (DW) for 24 h at 40°C, with the water bath being changed five times throughout the process. Dialysis was limited to 24 h to prevent excessive hydrolysis of the gelatin. However, this duration was sufficient to remove most of the methacrylic acid, which is the hydrolyzed form of methacrylic anhydride (See Supplementary Figure S1), from the synthesized GelMA. Upon completion of dialysis, the pH of the GelMA solution was adjusted to 7.4 using NaOH. Subsequently, the solution was transferred to petri dishes and allowed to solidify into a gel. Once solidified, the petri dishes were frozen at −20°C. Finally, freeze-drying (Christ, Alpha 2-4 LSCplus) was performed to isolate GelMA. A 100 g synthesis of GelMA took approximately 8–10 days to dry in the freeze-dryer. ^1^H-NMR spectroscopy affirmed the presence of the modification, evidencing a substitution degree of 94%.

### 2.2 Small molecular weight alginate preparation

Small molecular weight alginate (SMWA) was created by dissolving alginate from a commercial source (original alginate) (Sigma A1112) at a concentration of 20 mg/mL. The solution was continuously stirring for 3 days at 80°C, followed by dialysis in membranes with a MWCO of 3,500 Da (Spectra/Por^®^ 7, 132111). Afterwards, the alginate was collected from the dialysis membranes and freeze-dried. A 20 g synthesis of the SMWA took approximately 8–10 days to dry in the freeze-dryer.

### 2.3 Rhodamine-GelMA synthesis

Rhodamine was used to label GelMA following the protocol described by Ouyang et al. (2022). The pH of PBS was adjusted to 8.1 with NaOH. GelMA was dissolved in the pH-adjusted PBS at 10 w/v% at 50°C under stirring. Upon complete dissolution of the compound, N-hydroxysuccinimide (NHS)-Rhodamine (Thermo Fisher, 46406) was added to the dissolved GelMA at a concentration of 0.5 mg/mL. After 6 h of reaction at 50°C in the dark, the mixture was dialyzed in membranes with a MWCO of 12k-14k Da against DW at 40°C until no NHS-Rhodamine was detected in the dialysis water (5 changes approximately per day). After dialysis, the pH of the solution was adjusted to 7.4 using NaOH. After freeze-drying, the synthesized Rhodamine-GelMA was stored at −20°C, protected from light, until use.

### 2.4 Fluorescein-labeled alginate synthesis

Original alginate and SMWA were labeled with fluorescein based on adaptations of the protocols described by Dadoo et al. (2017), Strand et al. (2003). The alginate was dissolved in DDW at a concentration of 10 mg/mL. 1-Ethyl-3-(3-dimethylaminopropyl) carbodiimide (TCI, D1601), NHS (Sigma-Aldrich, 8.04518) and Fluoresceinamine Isomer I 99% (Thermo Fisher, 400770050) were subsequently added to reach a concentration of 0.308 mM, 0.308 mM and 0.0358 mM, respectively. The mixture was kept under stirring at room temperature (RT) (21°C–23°C) for 18 h. Afterwards, the alginate was first precipitated in ice cold acetone, followed by redissolving the precipitate and dialysis in membranes with a MWCO of 12k-14k Da against DW, until no Fluoresceinamine was visually detectable in the dialysis water. After dialysis, the pH of the solution was adjusted to 7.4 using NaOH, followed by lyophilization in the dark. Dried fluorescein-labeled alginate was stored at 5°C and protected from light until further use.

### 2.5 Alginate dissolution fraction determination

To determine the dissolution fraction, calcium-crosslinked SMWA gels of approximately 600 mg were weighed and freeze-dried immediately after crosslinking to determine the dry mass ( $M_{d0}$ ). Next, the dried SMWA gels were incubated in DDW or standard leaching solution (SLS), consisting of 55 mM sodium citrate (Sigma-Aldrich, W302600), dissolved in PBS (2.67 mM KCl, 1.47 mM KH_2_PO_4_, 137.93 mM NaCl, and 8.06 mM Na_2_HPO_4_-7H_2_O) (Gibco™, 14190), at RT for 20 min. Afterwards, the supernatant was removed and the gels were freeze-dried again to determine the second dry mass ( $M_{de}$ ). The dissolution fraction was determined from both masses using the following formula:

$$Dissolution\;fraction\;\left(\%\right)=100-\left(\frac{M_{De}}{M_{D0}}\right)*100$$

### 2.6 Photo-initiator synthesis

The photo-initiator lithium (2,4,6-trimethylbenzoyl) phenylphosphinate (Li-TPO) was synthesized according to a previously reported protocol (Markovic et al., 2015). Briefly, 109 mmol lithium bromide (Sigma-Aldrich, 746479) was dissolved in 150 mL butanone (Sigma-Aldrich, 360473) followed by the addition of 27.2 mmol of (2,4,6-trimethylbenzoyl)-phenyl-phosphinic acid ethyl ester (Lambson, SpeedCure TPO-L). The mixture was allowed to react for 24 h at 65°C. The resulting precipitate was separated through filtration, washed with petroleum ether (Chemlab, CL00.1601), and then dried under vacuum at RT.

### 2.7 Porogen fabrication

**FIGURE 1:**
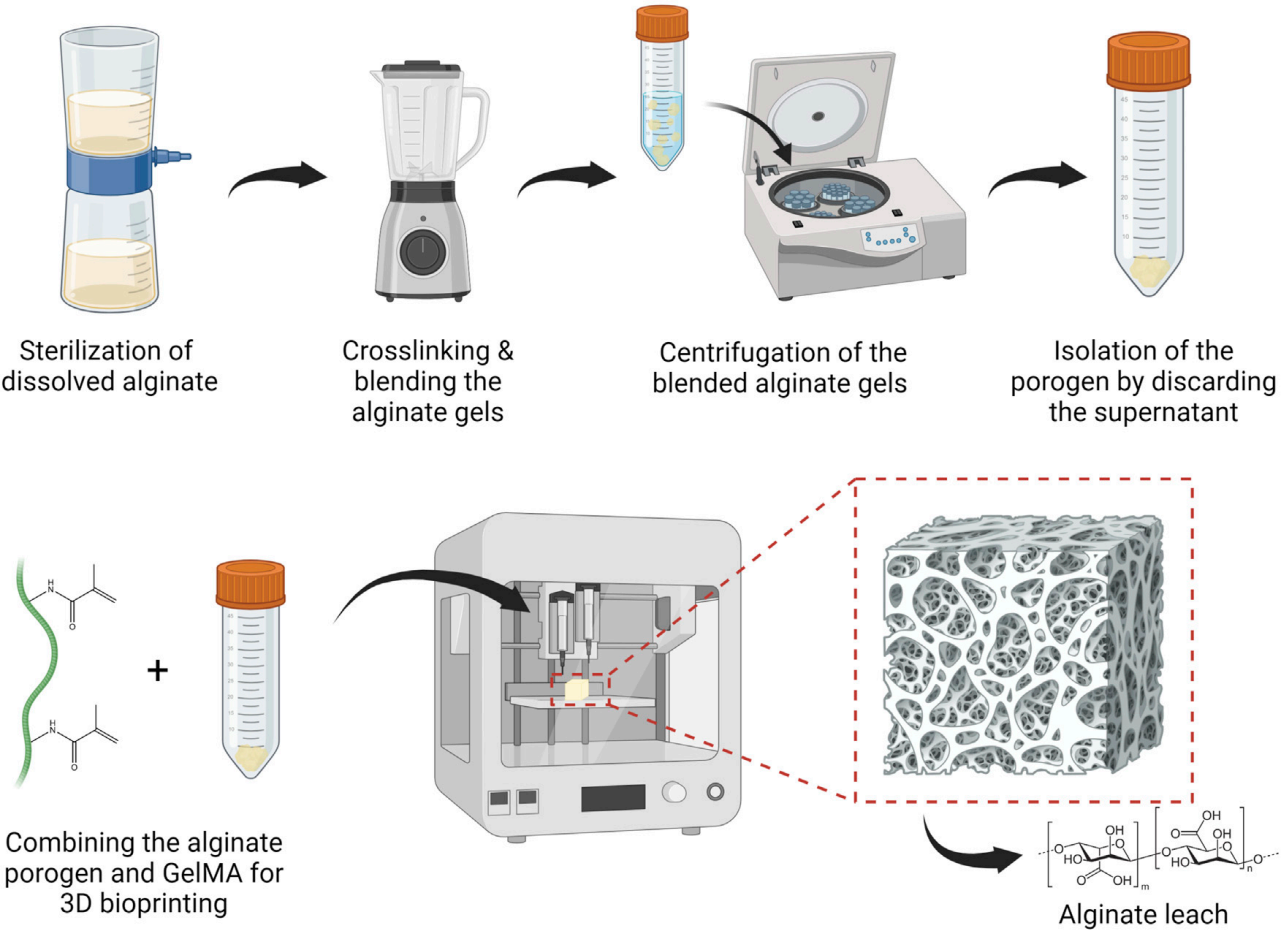
Standard operating protocol to fabricate and bioprint calcium-alginate porogen suspended in a hydrogel solution, followed by leaching the porogen from the 3D bioprinted structure. Figures were created with BioRender.com.

The porogen fabrication procedure is depicted in Figure 1. Original alginate and SMWA was dissolved in DDW at a concentration of 20 mg/mL. The solution was sterilized using a Steriflip-GP (Sigma-Aldrich, SCGP00525) vacuum filtration system and then mixed with a sterile CaCl_2_ (Sigma-Aldrich, C5670) solution at a concentration of 13 mg/mL. In the presence of divalent cations such as calcium, alginate forms an ionically crosslinked gel that is insoluble in water. The alginate gel and CaCl_2_ suspension was poured in a blender that was sterilized by intensive cleaning with 70% ethanol. The crosslinked product was then cut into small particles by the blender for 30 s. Subsequently, the mixed solution was transferred to 50 mL tubes and centrifuged for 5 min at 1,000 rpm. The supernatant was removed and the porogen was used directly for bioprinting or stored at 4°C.

### 2.8 Biomaterial preparation

Only sterilized biomaterials were used when involving cells. Biomaterials were sterilized by Steriflip-GP vacuum filtration. Freeze-dried GelMA was weighed and dissolved in sterilized DDW at a concentration of 10 w/v%. The amount of alginate porogen to be weighed was determined based on the mass of GelMA. For a porogen-GelMA ratio of 16:1, the amount of porogen needed was 16 times the amount of the dry mass of GelMA. Before combining the GelMA solution and porogen, 2 mol% of Li-TPO with respect to the photo-crosslinkable moieties in GelMA, was added to the GelMA solution.

### 2.9 Fluorescein-labeled alginate leaching from Rhodamine-GelMA

Fluorescein-labeled original alginate or SMWA was mixed with Rhodamine-GelMA in a 16:1 ratio. 2 mol% of Li-TPO with respect to the photo-crosslinkable moieties in GelMA was added. The mixtures were pipetted in between two parallel glass plates coated with Teflon release foil and separated by a 1 mm thick silicone spacer. The samples were physically crosslinked at 5°C for 15 min before applying 10 min UVA-irradiation (365 nm, 10 mW/cm^2^) from above. 3 mm diameter pieces were punched out, transferred to a 96 well plate and immersed in CO_2_ independent medium (Gibco, 18045-088) with a pH between 7.35 and 7.45, supplemented with 0.3 w/v% sodium azide (Sigma-Aldrich, S2002). These samples were then incubated in a 37°C incubator. Images before and after incubation in SLS were made with an inverted fluorescence microscope (Nikon Ti). When making timelapse images, the interval between images was 15 s.

### 2.10 Mechanical analysis of the scaffolds

GelMA and 16:1 porogen-GelMA were pipetted in between two parallel glass plates coated with Teflon release foil and separated by a 5 mm thick silicone spacer. Next, the samples were physically crosslinked at 5°C for 15 min before applying 10 min UVA-irradiation (365 nm, 10 mW/cm^2^) from above. Prior to mechanical analysis, the scaffolds were swollen to equilibrium at 37°C in CO_2_ independent medium (Gibco, 18045-088) with a pH between 7.35 and 7.45, supplemented with 0.3 w/v% sodium azide. The porogen-GelMA scaffolds were incubated in SLS for either 20 min or 2 days. All hydrogels tested in this assay were punched out to obtain cylindrical shapes with a diameter of 6.5 mm. Mechanical compression was performed with a model 5ST Bench-Mounted Universal Testing Machine (Tinius Olsen), equipped with a 25N load cell. The scaffolds were submitted to stress and strain at increasing levels and at a constant speed of 3 mm/min. The compression modulus was determined based on the slope of the linear region of the stress-strain curves using the following formula:

$$E=\frac{Compressive\;stress}{Axial\;strain}=\frac{Force/Area}{Compressed\;distance/Original\;height}$$

### 2.11 Cell culture

Human Umbilical Vein Endothelial Cells (HUVECs) (Thermo Fisher, C0035C) were cultured in culture falcons (VWR^®^, 734-2309, 734-2313, 734-2315) in endothelial cell growth medium 2 (EGM2) (Promocell, C-22011) enriched with 1 v/v% Penicillin/Streptomycin (Pen/Strep) (Sigma-Aldrich, P4333). Only HUVECs below or equal to passage 6 were used. Adipose derived stem cells (ASCs) (Lonza, PT-5006) were cultured in culture falcons in Dulbecco’s Modified Eagle Medium (DMEM) (Gibco™, 31966021), supplemented with 1% Pen/Strep and 10 v/v% Fetal bovine serum (FBS) (Gibco™, A3160801). Only ASCs below or equal to passage 15 were used.

### 2.12 Proliferation assay

The influence of the SLS and a higher concentration leaching solution (HCLS), consisting of 8x concentrated PBS and 220 mM sodium citrate, was tested on HUVECs. HUVECs were seeded in 96 well plates at a concentration of 3,000 cells per well and cultured in EGM2. After 1 day of incubation, the culture medium was removed, and the different leaching solutions were added for different time periods. After these different time periods, the leaching solutions were removed again, and the cells were again cultured in EGM2.

The cells were evaluated on day 0 right before adding the different leaching solutions, on day 1, 24 h after the cells were treated with the leaching solutions, and again on day 4. The cellular reducing environment, correlating to the total amount of viable cells, was evaluated using PrestoBlue™ Cell Viability Reagent (Invitrogen™, A13261). 100 μL of PrestoBlue reagent diluted at a ratio of 1:10 in culture medium was added to each well and incubated in the dark for 2 h. A Wallac 1420 Victor2 Microplate reader (Perkin Elmer) was employed to measure the fluorescence generated in the wells. The fluorescence of the same wells was monitored over time. The proliferation percentage for each well was determined using the formula provided below after background fluorescence was subtracted:

$$Proliferation\;\left(\%\right)=\frac{Fluorescence\;on\;days\;1\;and\;4\;after\;treatment}{Fluorescence\;before\;applying\;treatments}*100$$

### 2.13 3D bioprinting

#### 2.13.1 Basic operations

For 3D bioprinting, the screw extrusion-based REGEMAT BIOV1 Bioprinter (REGEMAT) was used. After loading the bioprinting cartridges with the bioink, they were allowed to set at 5°C for 30 min. Biomaterials printed at 5°C were printed directly after this incubation step. Biomaterials printed at RT (21°C–23°C) and 26°C were incubated a second time at the respective temperature for 30 min before starting the bioprinter. SmoothFlow Tapered Dispense Tips (Nordson EFD, 7018298) with an inner diameter of 410 µm were used as printing nozzle.

#### 2.13.2 Printability assessment

For the printability evaluation prints, the biomaterials were loaded into the syringes without resuspension of a cell pellet and visualized immediately after printing. The printability was evaluated according to a protocol described by Ouyang et al. (2016). This evaluation was performed by characterizing the shape of the pores in a bioprinted grid-like structure based on the theoretical pore characteristics determined by the CAD design. The CAD design was created on the REGEMAT3D Designer software and consisted of a strut printed perpendicular to create rectangular pores with dimensions of 2.8 × 2.3 mm^2^. The grids were printed on microscopy slides (Epredia™, J1800AMNZ). The pores were visualized under a phase-contrast microscope (Olympus BX43^®^). The area and perimeter of the pores were determined using ImageJ (Fiji). The Printability Rate (PR) was calculated with the following formula:

$$\mathrm{PR}=0.778*\;\frac{{Perimeter}^{2}}{Area*4*\pi }$$

#### 2.13.3 Sterile bioprinting of cell-laden biomaterials

All steps were performed inside a laminar flow cabinet. All components that came in direct contact with the bioink were sterilized by spraying them with 70% ethanol and allowing them to air-dry. This sterilization step was repeated twice. The cartridges were loaded with the cell-laden bioinks, set to temperature as described in Section 2.13.1, and inserted in the bioprinter. Once bioprinted, the scaffolds were incubated at 5°C for 5–10 min. Consequently, they were crosslinked by UVA-irradiation (365 nm, 5–10 mW/cm^2^) from above for 10 min. The prints were covered with an appropriate cell medium specific for each assay and incubated at 37°C in a humidified atmosphere containing 5% CO_2_ for 1 week. The medium was refreshed twice.

The design used for cell printing consists of 5 identical rectangular layers printed on top of each other, with each layer consisting of 8 parallel filament struts. The construct measures 6 × 9 × 2.25 mm^3^ (w × l × h).

### 2.14 Viability assay

For the viability assay, the HUVECs were encapsulated in GelMA and 16:1 SMWA porogen-GelMA. The cell pellet was resuspended in the biomaterial and photo-initiator solution, at a concentration of 4 million cells/mL. The cell-laden porogen-GelMA and GelMA were then bioprinted at 5°C and 26°C, respectively, and photo-crosslinked as described in Section 2.13.3. Subsequently, the scaffolds were resuspended in EGM2. 24 h later, the samples were incubated in SLS for 20 min at 37°C. Afterwards, the samples were resuspended again in EGM2.

The Live/Dead viability assay exploiting Calcein Acetoxymethyl ester (Ca-AM) (Invitrogen™, 15550597) and Propidium Iodide (PI) (Invitrogen™, BMS500PI) was used to assess the viability of the cells. GelMA scaffolds were rinsed two times with PBS. A working solution of 0.2 μg/mL Ca-AM and 0.2 μg/mL PI was added for 10 min in the dark at RT. The scaffolds were examined using an inverted fluorescence microscope (Nikon Ti). A green fluorescent protein (GFP) and Tetramethylrhodamine isothiocyanate (TRITC) filter were used to visualize living and dead cells, respectively. Pictures were collected as Z-stacks and examined with ImageJ (Fiji). Viability was calculated with the following formula:

$$Viability\;\left(\%\right)=\frac{Living\;cells\;}{Living\;cells+Dead\;cells\;}*100$$

### 2.15 3D VEGF bioassay

After thawing, the VEGF responsive cells (KDR/NFAT-RE HEK293) (Promega, GA 2001) were directly encapsulated in the biomaterials at a concentration of 1,000,000 cells/mL. After bioprinting porogen-GelMA and GelMA at 5°C and 26°C, respectively, and photo-crosslinking the scaffolds as described in Section 2.13.3, the scaffolds were resuspended in DMEM and supplemented with 10% FBS. One hour later, the samples were submerged in SLS for 20 min at 37°C. Afterwards, they were incubated in DMEM, supplemented with 10% FBS and 100 ng/mL human VEGF165 (Peprotech, 100-20-10UG). 17 h later, a D-luciferin (Gold Biotechnology, LUCK-1G) solution in PBS was added to each well at a final concentration of 1.5 mg/mL. After 10 min of incubation in the dark, luminescence was measured with a Wallac 1420 Victor2 Microplate reader (Perkin Elmer).

### 2.16 Vascularization assays

HUVECs and ASCs were isolated from their culture falcons and counted. For the vasculogenesis assay, a total cell count of 4 million cells in a ASC/HUVEC ratio of 1.7 were encapsulated per ml biomaterial. For the angiogenesis assay, ASCs and HUVECs were cocultured at a ratio of 1,000:5,000 cells in EGM2 cell medium (Promocell) in Nunclon™ Sphera™ 96-Well low attachment plates (Thermo Fisher, 174925) at 37°C and 5% CO_2_. The next day, they were isolated from the well plate as spheroids. A spheroid pellet was created through centrifugation and was resuspended in the GelMA or Porogen-GelMA biomaterials. Per biomaterial composition, spheroids from two full 96-Well low attachment plates were encapsulated. After bioprinting the cell-laden porogen-GelMA and GelMA at 5°C and 26°C, respectively, and photo-crosslinking the scaffolds as described in Section 2.13.3, the scaffolds were resuspended in EGM2 cell medium. 24 h later, the samples were submerged in SLS for 20 min at 37°C. Afterwards, the vasculogenesis and angiogenesis samples were resuspended in EGM2 and EGM2, supplemented with 100 ng/mL recombinant human VEGF165, respectively.

Z-stack images made with a x/y/z voxel size of 2.45 × 2.45 × 2.825 μm^3^/Voxel by a Nikon A1R HD confocal microscope were deconvoluted using Deconvolutionlab2 in ImageJ (Fiji). Deconvoluted Z-stack images of by vasculogenesis created vascular networks were analyzed with VesselVio software. Isolated segments shorter than 100 µm and end point segments shorter than 50 µm were removed. 3D Vasculogenesis images were created by importing the deconvoluted Z-stack in Fluorender. Sprouting angiogenesis from the spheroids was analyzed by tracing the total length of vascular sprouts on the deconvoluted Z-stack images in ImageJ. Total spheroid spread area was calculated by connecting the farthest reaching points of the individual sprouts with straight lines and calculating the area of the created polygon. The 3D angiogenesis images were created by importing a deconvoluted Z-stack in 3D viewer on ImageJ (Fiji).

### 2.17 Whole-mount immunohistochemical staining

The scaffolds were fixed by immersion in formaldehyde solution 4% (VWR®, 4078.9010) for 15 min at RT. The scaffolds were then rinsed twice with PBS. Blocking was done by covering the samples with a 2% Bovine serum albumin (BSA) (Sigma-Aldrich, A9418) solution for 1 h at RT. Afterwards, the scaffolds were rinsed twice with PBS and incubated overnight at 5°C with a 1:50 fluorescein isothiocyanate conjugated CD31 (PECAM-1) antibody (eBioscience™, 11-0319-42, RRID: AB_2043835) (1:50 in a 2% BSA solution). To stain VE-Cadherin junctions, the samples were permeabilized before blocking by incubating them for 5 min with 0.1 v/v% Triton™ X-100 (Sigma-Aldrich, T8787) in PBS, followed by two times rinsing in PBS. After blocking and washing in PBS, the samples were allowed to react overnight at 5°C with a CD144 (VE-cadherin) monoclonal antibody (eBioscience™, 14-1449-82, RRID: AB_467495) (1:50 in a 1% BSA solution). Subsequently, the scaffolds were rinsed twice with PBS and incubated overnight with an Alexa Fluor™ 555 conjugated goat anti-mouse IgG secondary antibody (Thermo Fisher, A-21422, RRID: AB_2535844) (1:100 in a 1% BSA solution). Lastly, the scaffolds were resuspended in a 2 μg/mL Hoechst 33258 (Thermo Fisher, H1398) solution in PBS for nuclear counterstaining and examined using the Nikon A1R HD confocal microscope.

### 2.18 CellTracker red staining

For confirmation of successful whole-mount immunohistochemical staining of the vascular structures embedded in hydrogels, HUVECs were also stained with CellTracker™ Red CMTPX Dye (Thermo Fisher, C34552). The dye was prepared by dissolving 50 μg CellTracker™ Red powder in 8 μL dimethylsulfoxide, and from this solution, 2.5 μL was combined with 4 mL DMEM. The CellTracker Red–DMEM solution was added to the HUVECs’ culture flask after aspirating the culture medium and rinsing twice with PBS. Subsequently, the flask was incubated for 45 min at 37°C, in the presence of 5% CO_2_. After the incubation, the staining solution was removed and the cells were again covered with culture medium and incubated for at least 1 h at 37°C and 5% CO_2_ before further use.

### 2.19 Statistics

All data were analyzed using GraphPad Prism 8.0.2. When there were only two groups of data to be compared, an unpaired Student’s t-test was conducted. When there were more than two groups to be analyzed, a one-way ANOVA followed by a Tukey’s multiple comparison test was performed. Statistical significance was stated as *p* ≤ 0.05.

## 3 Results and discussion

### 3.1 Porogen characterization

Alginate was explored as a porogen because it is considered a cost-effective, biocompatible material that is easy to handle (Sun and Tan, 2013). However, the commercial alginate source used in this article still contains pro-inflammatory contaminants. For use in animal and human studies, it is advisable to extensively purify the alginate before implantation to prevent immunological reactions (Paredes Juárez et al., 2014). A procedure for easily fabricating low-cost alginate porogen has been developed. This procedure is depicted in Figure 1 and described in Section 2.7.

To ensure that cells embedded in the porogen-GelMA mixtures can profit optimally from the increased porosity, a dissolution condition needed to be found that permits the porogen to dissolve and leach from the hydrogel within a reasonable time frame without having substantial impact on cell viability and behavior. A screening design, generated with design of experiments software, was conducted to assess the effectivity of different conditions to rapidly dissolve calcium-crosslinked alginate (See Supplementary Data 1.1 and Supplementary Figure S2).

It was concluded that a solution of sodium citrate in PBS at a concentration of 55 mM was the most effective in completely dissolving 600 mg alginate gels, made with calcium-crosslinked alginate from a commercial source (original alginate), within 20 min. Both the phosphate from the PBS and the sodium citrate are able to capture the calcium ions from the crosslinked alginate, which results in the dissolution of the alginate (Groboillot et al., 1994; Wu et al., 2016). The combination of PBS and 55 mM sodium citrate will be referred to as the standard leaching solution (SLS).

**FIGURE 2:**
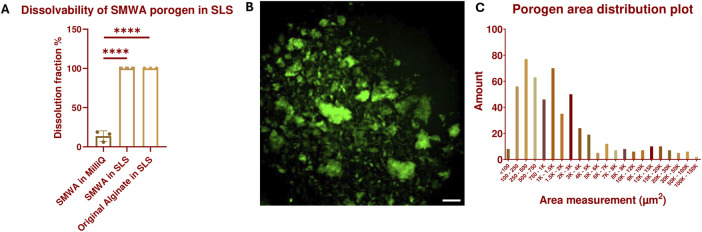
Dissolvability and size characterization of the SMWA porogen: **(A)** Confirmation of the 100% dissolvability of SMWA gels, with an approximate weight of 600 mg, after 20 min of incubation in SLS. N = 3. **** = *p* < 0.0001. The data for the original alginate in SLS are generated by the Design of Experiments shown in Supplementary Data 1.1 and Supplementary Figure S2. **(B)** Fluorescein-labeled SMWA porogen suspended in DDW. Scale bar = 250 µm. **(C)** Distribution plot of the individual porogen particles based on their measured area from **(B)**. N = 533.

However, as seen in Supplementary Figure S3, leaching out the original alginate porogen from a GelMA hydrogel to the surrounding medium through diffusion over 20 min and even during 2 days of incubation in the SLS solution was ineffective. To improve the porogen leaching from the hydrogel structure, a small molecular weight version of the original alginate (SMWA) was developed. This was created by continuously stirring dissolved original alginate for 3 days at 80°C, which degraded the alginate into shorter molecular chains. The reduction in chain length, and consequently the molecular weight of the heated alginate in comparison to the original source was confirmed by GPC analysis (see Supplementary Data 1.2; Supplementary Figure S4; Supplementary Table S1). The weight average molecular weight of the SMWA is 16,190 g/mol, while that of the original alginate is 166,700 g/mol. In Figure 2A, it is shown that large SMWA gel pieces of approximately 600 mg were also dissolvable in SLS in less than 20 min, which is significant in comparison to the negative control in DDW. Figure 2B shows an epifluorescence image of fluorescein-labeled SMWA porogen. The image indicates that the porogens do not have a uniform shape. Figure 2C depicts the distribution of porogen sizes. From this graph, it is clear that the porogen consists of a heterogeneous collection of different sizes of calcium-crosslinked SMWA gels. All together this means that the pores created in the hydrogel by the SMWA will be of varying shapes and sizes. These heterogeneous porous structures are not necessarily a disadvantage for the vascular development in hydrogel structures. Mehdizadeh et al. used computational models to model vascular ingrowth in porous scaffolds. They concluded that scaffolds with heterogeneous pore sizes are more favorable for vascular ingrowth in comparison to homogenously shaped pores in scaffolds with an identical total volume of pores (Mehdizadeh et al., 2013).

**FIGURE 3:**
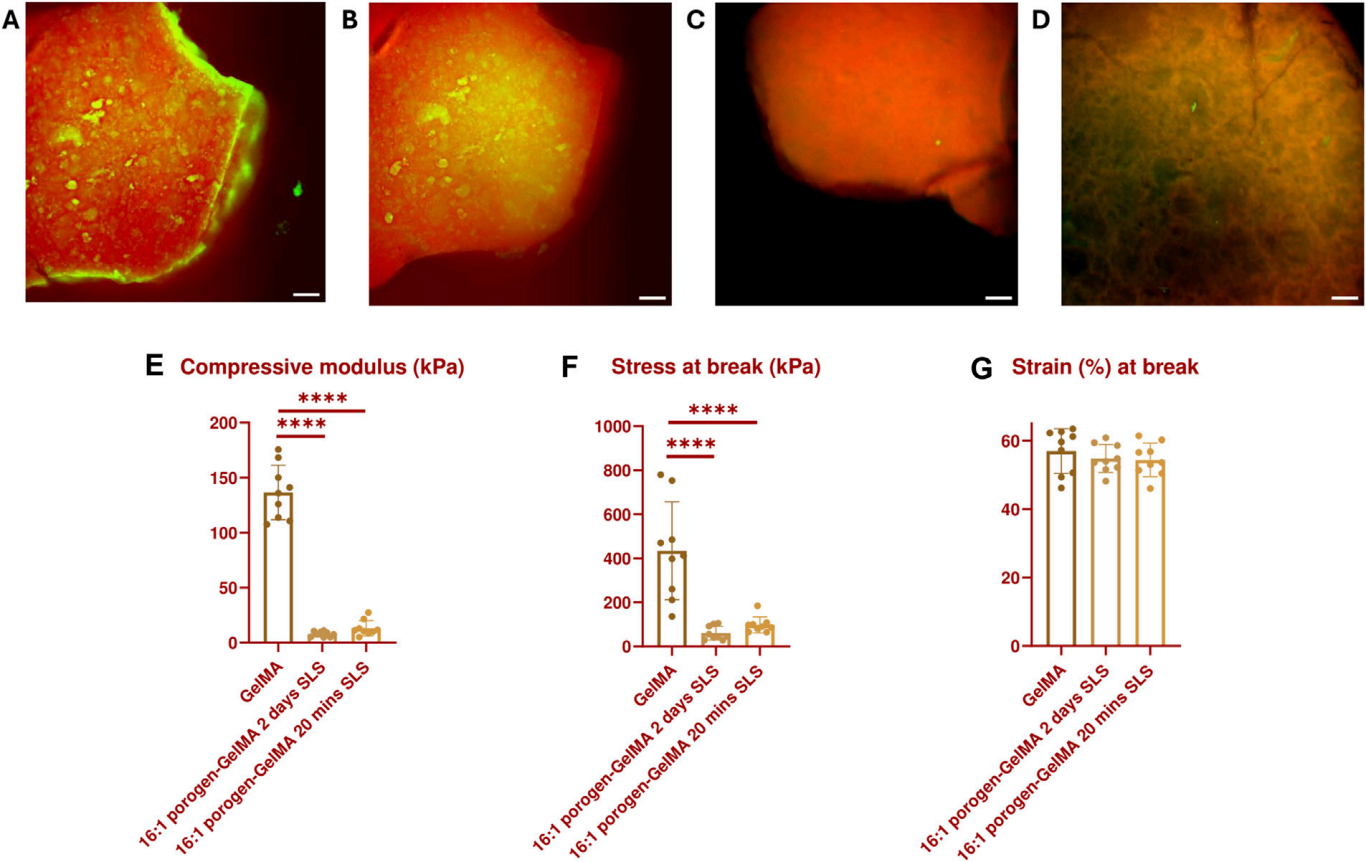
GFP- and TRITC-filtered composite images of a 16:1 fluorescein-labeled SMWA porogen and Rhodamine-GelMA blend: **(A)** Before incubation in SLS; **(B)** After incubation in SLS for 20 min; **(C)** After incubation in SLS for 48 h; **(D)** after incubation in SLS for 48 h, imaged from a different angle to better visualize the porous structures. Scale bars = 250 µm. Mechanical properties characterization: **(E)** Compressive modulus, **(F)** Stress at break, and **(G)** Strain at break of GelMA and 16:1 porogen-GelMA, incubated for 20 min or 2 days in SLS. N = 9.

A leaching test was performed by encapsulating fluorescein-labeled SMWA porogen in Rhodamine-GelMA in a 16:1 ratio. Test samples were cylindrical, with a diameter of 3 mm and a height of 1 mm. After 20 min of leaching in SLS, there was a significant reduction of porogen in the hydrogel (see Figures 3A, B and Supplementary Movie 1). However, not all the porogens were able to leach out within this time period and some porogen seemed to travel to the center of the hydrogel instead of outwards. After continuing incubation in SLS for 2 days, the porogen was able to leach completely from the hydrogel (see Figures 3C, D), in contrast to the original alginate porogen (see Supplementary Figure S3). This suggests improved leaching of the SMWA compared to the original alginate. For this reason, the SMWA porogen will be used as the standard porogen throughout this article.

In addition, the mechanical properties of the 16:1 SMWA porogen-GelMA hydrogel were investigated (see Figures 3E–G). No statistically significant difference in mechanical properties was recorded between porogen-GelMA hydrogels that were incubated in SLS for 20 min and 2 days. However, the compression moduli of the porogen-GelMA groups, 13.2 kPa ± 2.4 (20 min SLS) and 7.8 kPa ± 2.4 (2 days SLS), were significantly lower than the compression modulus of GelMA, 136.6 kPa ± 24.7. Stress at break was also significantly lower for the porogen-GelMA groups in comparison to GelMA. By contrast, no statistically significant difference was recorded between the groups in terms of the strain at break.

### 3.2 Bioprintability

**FIGURE 4:**
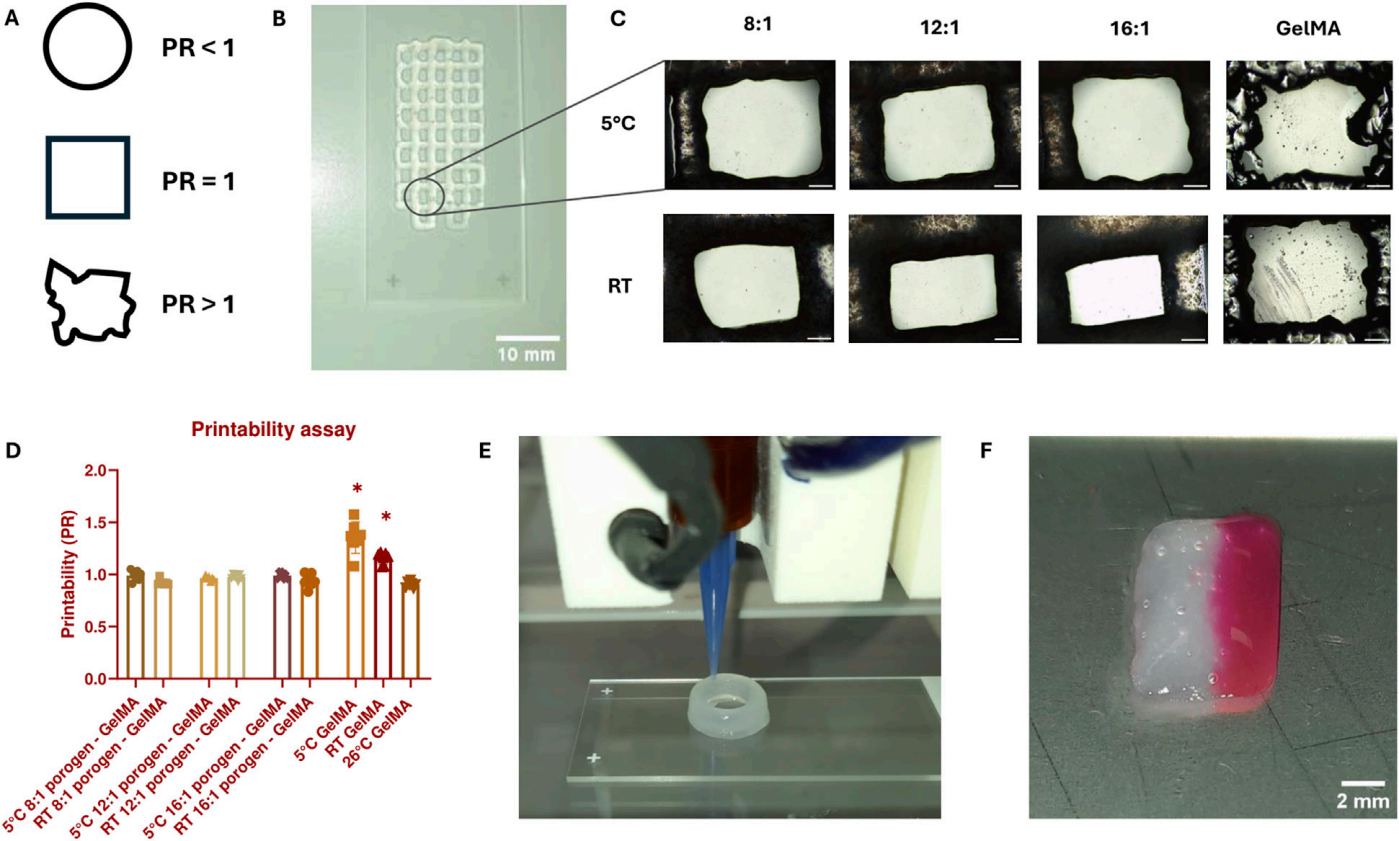
**(A)** Visual representation of the pore shape according to PR. **(B)** Macroscopic image of a scaffold with rectangular pores used for PR-calculations, bioprinted with 16:1 SMWA porogen-GelMA. Scale bar = 10 mm. **(C)** Close-up images of the printed pores used to calculate the PR. Scale bar = 500 μm. **(D)** Graph representing the PR for GelMA and porogen-GelMA bioprinted at different temperatures. The asterisks indicate a statistically significant difference between the PR of the bioprinted pores and a perfect pore with a PR = 1. N = 7. **(E)** Image of the Regemat BioV1 bioprinting the 16:1 SMWA porogen-GelMA in the shape of a hollow cylindrical structure. **(F)** Multimaterial bioprinting of 16:1 SMWA porogen-GelMA on the left side of the cuboid and Rhodamine-GelMA on the right side. Scale bar = 2 mm.

The combination of GelMA with SMWA-porogen needs to show sufficient shape-fidelity to replicate the designed CAD model and to make precise multimaterial bioprinting possible. To semi-quantify the printability of a biomaterial, the ability of the bioprinter to accurately recreate rectangular pores using a specific bioink composition was investigated. The PR reflects the shape of a printed pore (see Figure 4A). Pores that are nearly circular will have a PR less than 1, while more irregularly shaped pores will have a PR greater than 1. A pore that almost exactly matches the rectangular pore dimensions will have a PR very close to 1. Figure 4B shows a top-view macroscopic image of the printed filament struts with rectangular pores. Close-up images of the printed pores are depicted in Figure 4C. The printability of GelMA and porogen-GelMA in different ratios (i.e., 8:1, 12:1, and 16:1) is illustrated in Figure 4D. Regarding the porogen-GelMA, conditions printed at 5°C and RT were statistically insignificant to a control condition with a simulated prefect PR of “1”, thereby proving bioprintability. For GelMA, the best bioprintability was achieved at 26°C. The PR of GelMA printed at 5°C and RT was significantly higher than the control condition, which signifies an overgelated state. To prove the applicability of the porogen-gelMA combination for different designs, Figure 4E shows the bioprinting of a hollow cylindrical structure using the 16:1 porogen-GelMA bioink. In addition, in Figure 4F, an image of a cuboid is shown, printed on one side with porogen-GelMA (left) and Rhodamine-GelMA on the other side (right), showcasing its compatibility towards multimaterial bioprinting.

As illustrated in Figure 2C, the largest detected SMWA porogen has a surface area between 100,000 and 150,000 μm^2^. For a perfect circle of this size, this would mean that the diameter is between 357 and 437 µm. These big sized porogens could easily clog the bioprinting nozzle, which has a diameter of 410 µm. However, this was not observed during bioprinting. Most likely, thanks to processing the alginate into SMWA, the alginate gels can break easily into multiple fragments with limited amount of applied pressure, which assures a solid and uninterrupted flow through the needle.

### 3.3 Biocompatibility of the porogen leaching and 3D bioprinting

After proving the bioprintability of the SMWA porogen, the next step was to assess the impact of the porogen leaching and bioprinting on cellular viability. Complete leaching from a GelMA hydrogel was achieved after 2 days in standard leaching solution (SLS), which is not viable for encapsulated cells. 20 min incubation in a 55 mM citrate solution has been used in literature to dissolve cell-laden alginate gels (Wu et al., 2016). However, as seen previously, the leaching of the alginate porogen during 20 min in SLS was incomplete. Diffusion from the hydrogel to the surrounding medium over 4 days, following only 20 min of incubation in SLS on the first day, was not sufficient to leach out all the porogen from the 16:1 SMWA porogen-GelMA blends (see Supplementary Figure S5). It is hypothesized that the citrate and phosphate do not have sufficient time to reach and dissolve the porogens more deeply embedded in the hydrogel. Therefore, to maximize the leaching from porogen-GelMA mixtures and create the highest possible porosity, a longer leaching time of 40 min and a higher concentration of PBS and sodium citrate were explored. To confirm the feasibility of these leaching conditions, the potential negative effects on the cells were evaluated first.

**FIGURE 5:**
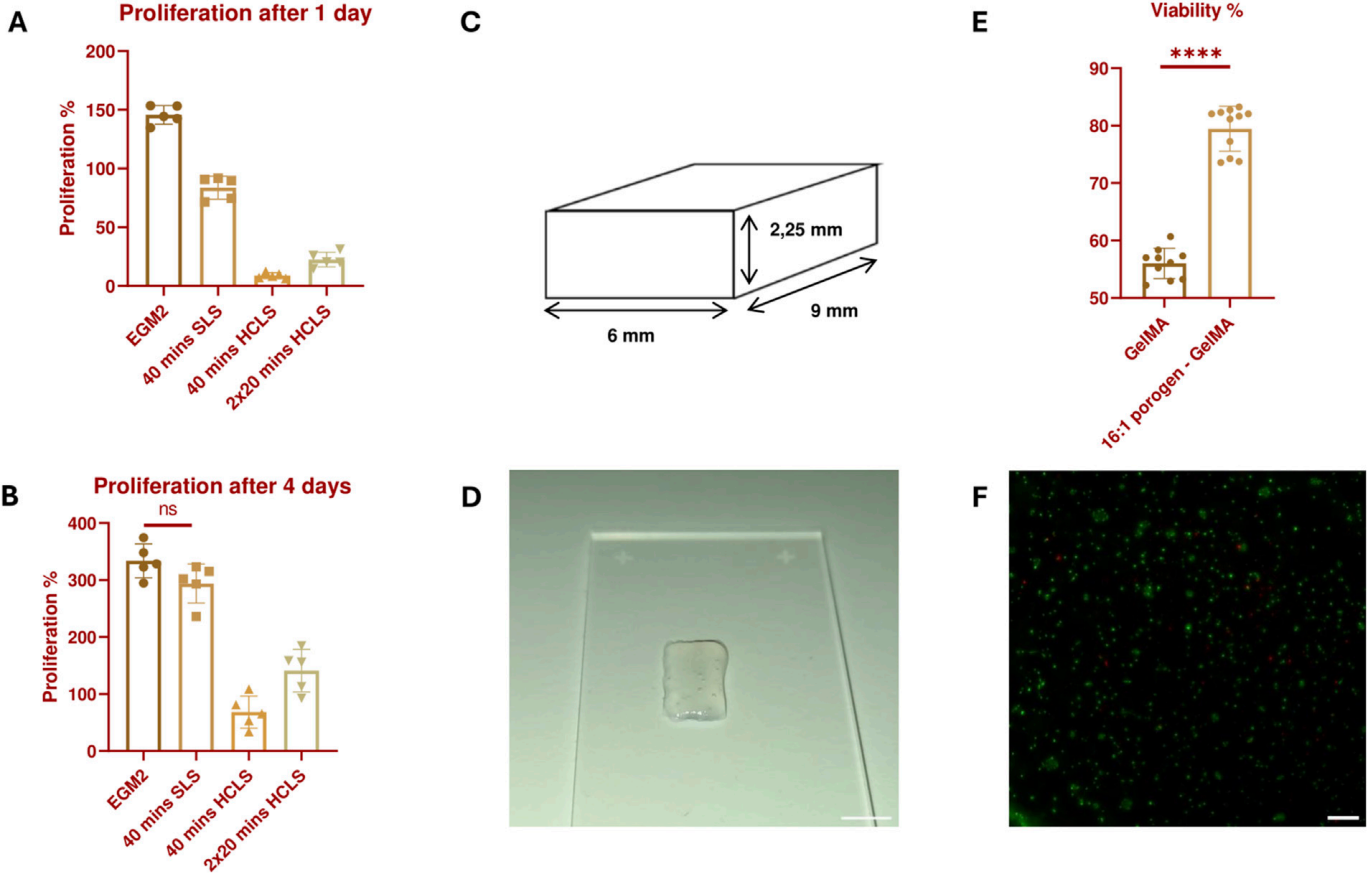
Proliferation percentage of HUVECs 24 h **(A)** and 4 days **(B)** after treatment with the different leaching solutions. All results are statistically significant (*p* ≤ 0.05) unless stated otherwise. N = 5. **(C)** 3D Design of a small solid structure constructed with adjacently printed struts with no space in between. **(D)** Image of a bioprinted small solid structure with a 16:1 SMWA porogen-GelMA bioink. Scale bar = 5 mm. **(E)** Live/Dead viability analysis of bioprinted HUVECs, encapsulated in GelMA and 16:1 SMWA porogen-GelMA. **** = *p* < 0.0001. N = 9. **(F)** Microscopic image of the Live/Dead staining of the encapsulated HUVECs in 16:1 SMWA porogen-GelMA. Green = living cells, Red = dead cells. Scale bar = 250 µm.

A comparison was made between the SLS and a higher concentration leaching solution (HCLS), consisting of 8x concentrated PBS and 220 mM sodium citrate. In a HUVEC proliferation assay, the HCLS seemed to have a significant negative impact on the HUVECs (see Figures 5A, B). Even with 1 hour of incubation in culture medium between two times 20 min incubation in SLS, the overall survival and proliferation on days 1 and 4 were still significantly lower in comparison to the HUVECs cultivated without leaching step and the 40 min in SLS. It is hypothesized that the non-isotonic nature of the higher concentration leaching method is the cause of the negative results observed under these conditions and that this, even for a short period, can be detrimental to the HUVECs. However, even with the less hypertonic SLS, the 40-min leaching condition had a negative effect on the cells. Although the HUVECs matched the proliferation level of the positive control after 4 days, their proliferative ability after 1 day was lower than 100%, which signifies a loss of cells compared to the cell population before treatment. Moreover, the proliferation percentage after 1 day was significantly lower compared to the HUVECs cultivated without the leaching step. This early reduction in cell viability and proliferation can impact the ability of ECs to form blood vessels and cannot be neglected. Therefore, despite possible incomplete leaching of the porogen from the hydrogel, the usage of the SLS was preferred over the HCLS and the duration of incubation in SLS was fixed to 20 min in the subsequent experiments.

A follow-up viability experiment was conducted on 3D bioprinted structures with HUVECs encapsulated in a 16:1 SMWA porogen-GelMA mixture. This ratio was used to test the effect of a maximum amount of incorporated porogen on the cells. The potential negative effects of suspending the cells in the DDW-GelMA solution, the 30 min incubation at 5°C, the mechanical stresses created during the bioprinting of cells with porogen, and the leaching of the porogen from the hydrogel, on the ECs were further investigated. In contrast to the bioprintability test in Section 3.2, the printed constructs did not contain bioprinted pore structures (see Figures 5C, D). The latter was to assess whether the porogen as such was sufficient to improve cell viability in the hydrogel construct. Live/Dead analysis after 7 days of culture indicated that the use of a porogen in the bioink significantly enhances the HUVECs’ viability (see Figures 5E, F). Although the porogen did not completely leach from the hydrogel after 20 min incubation in SLS, it already provided the scaffold with a sufficient increase in porosity as the viability of the encapsulated cells was enhanced significantly. As this condition gave favorable results, the subsequent experiments encompassed the use of the SMWA porogen and GelMA in a 16:1 ratio while the porogen was leached through incubation in SLS for 20 min.

### 3.4 Effect of the porogen on growth factor signaling

**FIGURE 6:**
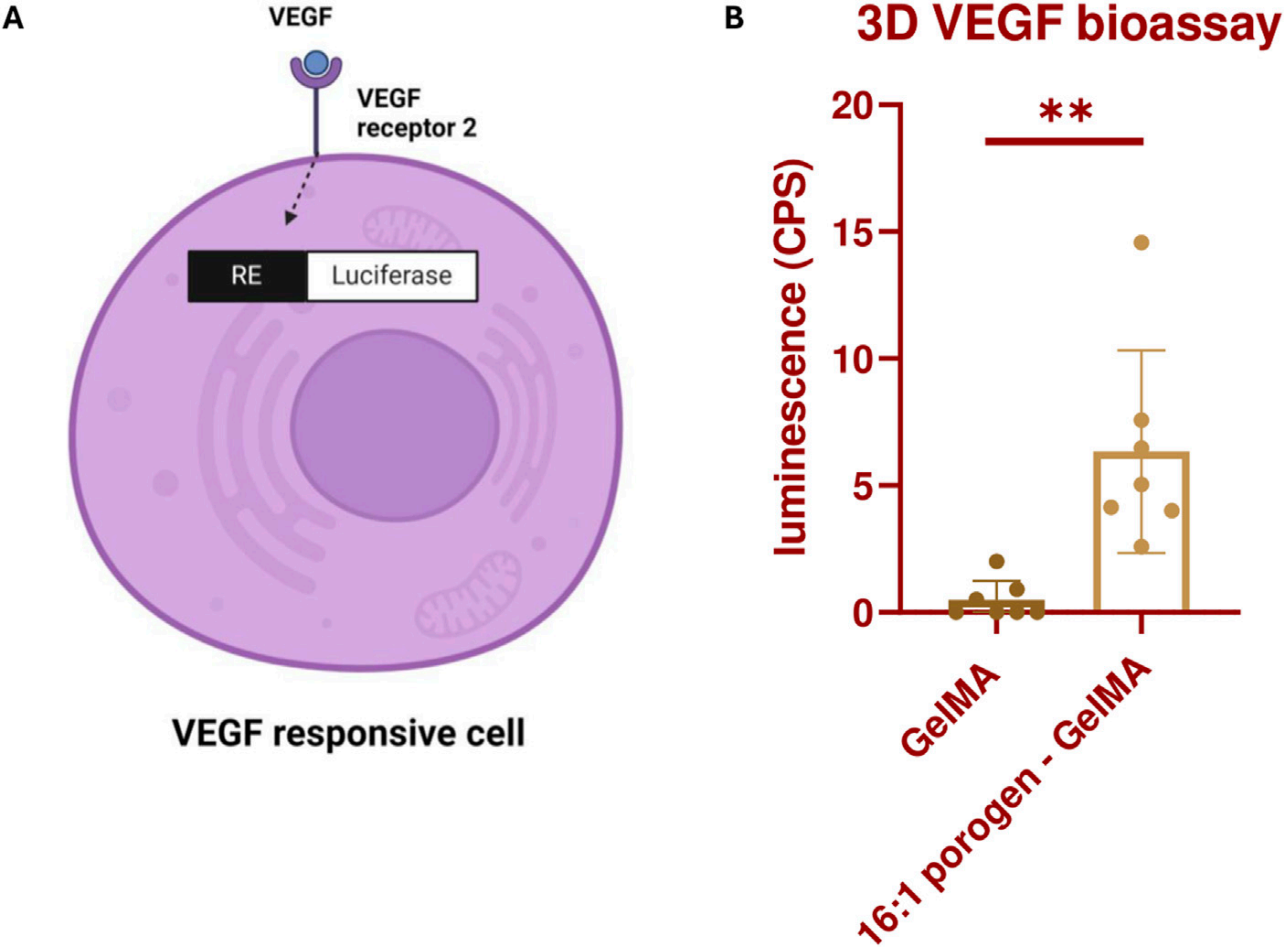
**(A)** Illustration depicting the production of luciferase by KDR/NFAT-RE HEK293 cells through the activation of the VEGFR2 by VEGF. The figure was created with BioRender.com. **(B)** Luminescence produced by the bioprinted KDR/NFAT-RE HEK293 cells, embedded in GelMA or 16:1 SMWA porogen-GelMA, after 17 h of exposure to recombinant human VEGF. N = 6. ** = *p* < 0.01.

To explain why the viability in the previous assay was increased and how the porous structure can aid in vascular development, we investigated whether vascular growth factors are able to reach the ECs better in a SMWA porogen-GelMA hydrogel compared to a bulk GelMA scaffold. To this end, VEGF responsive cells were encapsulated in GelMA with and without porogen and bioprinted in the same shape as used in the viability assay (see Figures 5C, D). The VEGF responsive cells were modified to include the NFAT response element upstream of Luc2P and to express VEGF Receptor 2 (VEGFR2) on its cell membrane. When VEGF interacts with the VEGFR2 on these cells, it triggers internal signaling that leads to production of luciferase mediated by the NFAT response element (see Figure 6A). Upon addition of D-luciferin, the luciferase converts the D-luciferin to light, which can be quantified. After 17 h of incubating the bioprinted structures in culture medium supplemented with 100 ng/mL VEGF, the luciferase activity of the cells was measured. The data illustrated in Figure 6B suggest that VEGF more effectively activates the VEGFR2 in the porogen-GelMA bioink compared to GelMA. This signifies that the growth factor is able to diffuse more freely through the porogen-GelMA biomaterial towards the cells, as is required for physiological vascular development.

### 3.5 Microvascular 3D bioprinting

To assess the potential of the SMWA porogen-GelMA to be used for microvascular bioprinting, first, the encapsulation of a vascular coculture of single cells was pursued, which was assessed for its ability to form a vascular network through vasculogenesis. Single cells of only HUVECs were not able to form blood vessels in the GelMA or porogen-GelMA mixture (see Figure 5F). However, as reported by various authors in literature, the combination of the ECs with mesenchymal stem cells enhances the formation of vascular networks (Jain, 2003; Chen et al., 2012).

**FIGURE 7:**
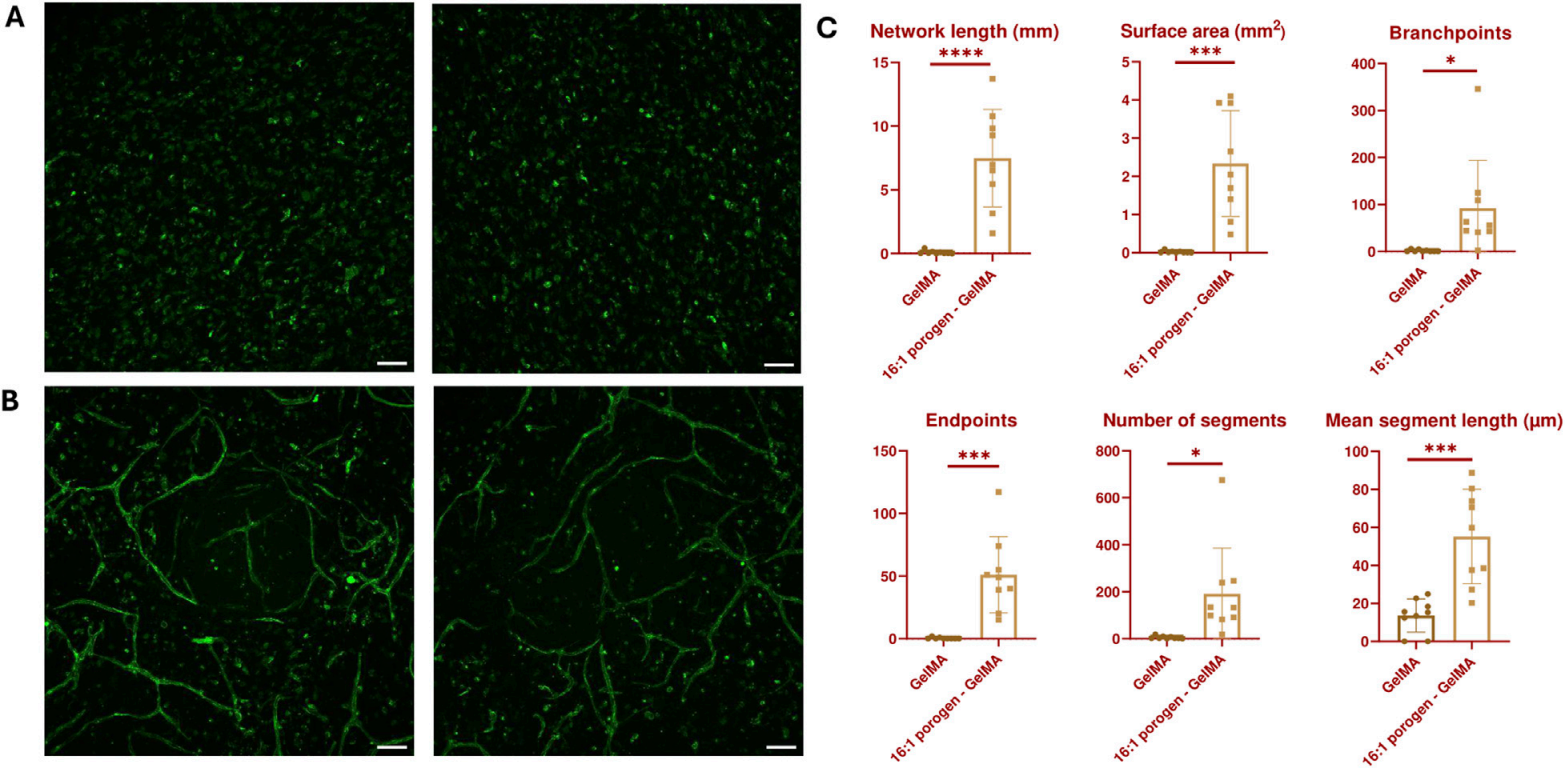
Maximum intensity Z-projection of CD31-stained vascular networks created through vasculogenesis in **(A)** GelMA and **(B)** 16:1 SMWA porogen-GelMA. Scale bar = 100 µm. **(C)** VesselVio analysis of distinct features of the vascular networks created through vasculogenesis in bioprinted GelMA and 16:1 SMWA porogen-GelMA scaffolds. N = 9. *, ***, **** = *p* < 0.05; 0.001; 0.0001, respectively.

In the following experiment, ASC and HUVEC cocultures were encapsulated in GelMA and in porogen-GelMA solutions. These bioinks were 3D bioprinted in the same shape as described in Figures 5C, D. A CD31 immunohistochemical staining was performed on day 7. Images of this staining are depicted in Figure 7. The fluorescent antibody was able to reach the cells at the lowest depth of the images taken from the GelMA group, which excludes possible bias from insufficient staining (see Supplementary Figure S6). From Figures 7A, B, it is observable that the addition of porogen improved the vascularization of the hydrogel structures. Additionally, the vessel structures were examined by the open-source software “VesselVio”, which allowed analysis of a 3D Z-stack image (Bumgarner and Nelson, 2022). Six different parameters (total network length, surface area, branchpoints, endpoints, number of segments and mean segment length) were deemed relevant and are depicted in Figure 7C. All six parameters were significantly higher for the porogen-GelMA blend in comparison to GelMA without porogen.

**FIGURE 8:**
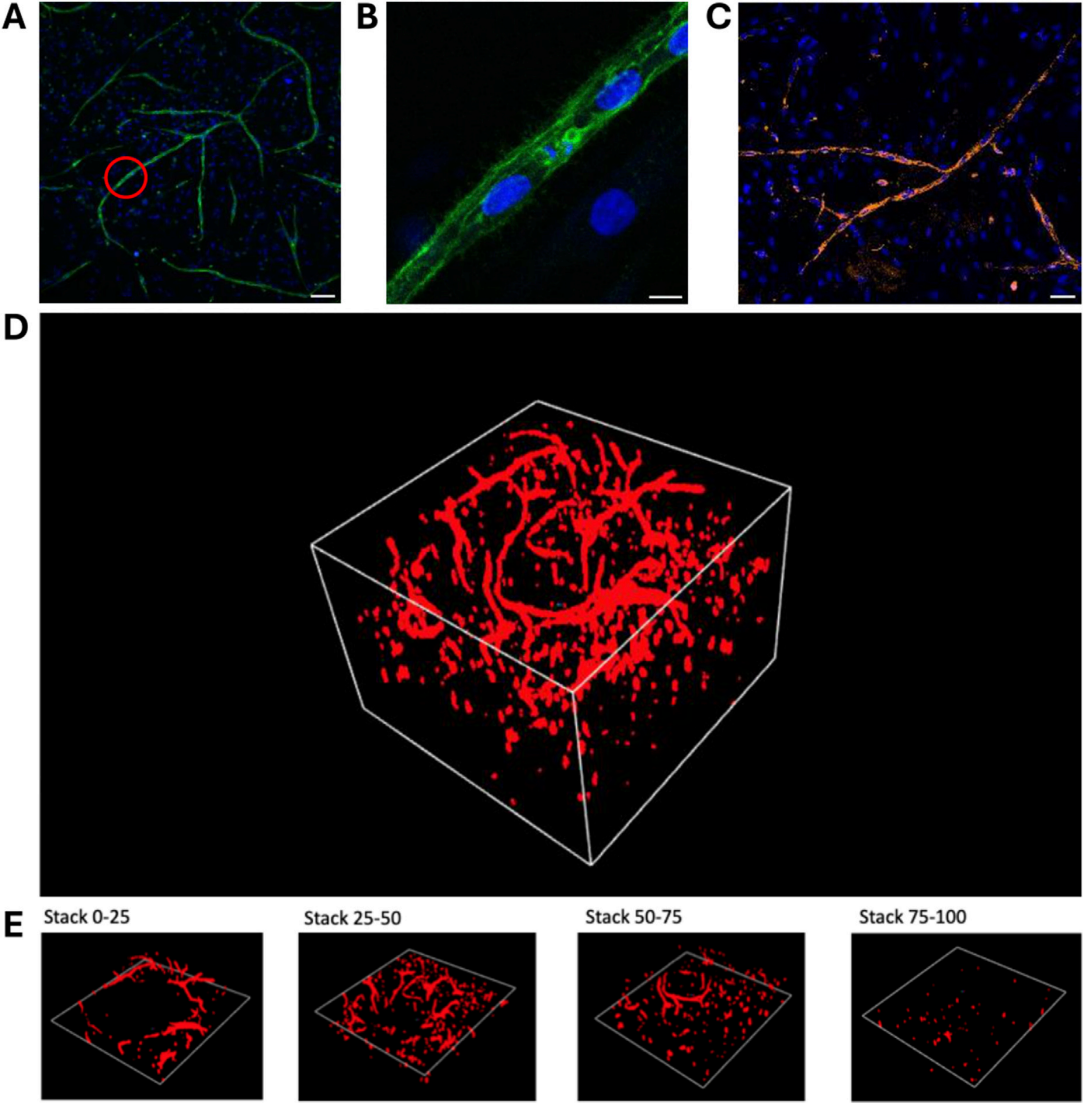
Immunohistochemical staining and image processing of vascular networks created through vasculogenesis after 7 days of culture, embedded in a 16:1 SMWA porogen-GelMA scaffold: **(A,B)** CD31 and Hoechst nuclear staining. The red circle in **(A)** indicates the region that is zoomed in on in panel **(B)**. Scale bar of **(A)** and **(B)** = 100 and 10 μm, respectively. **(C)** CD144 and Hoechst nuclear staining. Scale bar = 50 µm. **(D)** 3D image of a full CD31-stained Z-stack with a total Z-size of 305 µm (108 slices). **(E)** 3D images of every following ¼ of the Z-stack presented in **(D)**, bottom to top, respectively.

Figures 8A, B show vascular structures created by vasculogenesis, with a zoom-in on one of the main branches. The ECs’ nuclei are aligned between the stained CD31 junctions. A staining for CD144 was performed to demonstrate that the ECs in the fabricated vascular network also contain VE-Cadherin adherens junctions, and thus closely mimic native blood vessels (Figure 8C). A 3D image, depicted in Figure 8D, was made of one of the vascular networks in the porogen-gelMA scaffolds presented in Figure 7. In Figure 8E, distinct sections of the 3D image can be observed. These images confirm that the coculture can grow in a three-dimensional space within the hydrogel, mimicking its behavior in the native ECM, rather than merely forming a 2D monolayer on top of the scaffolds.

**FIGURE 9:**
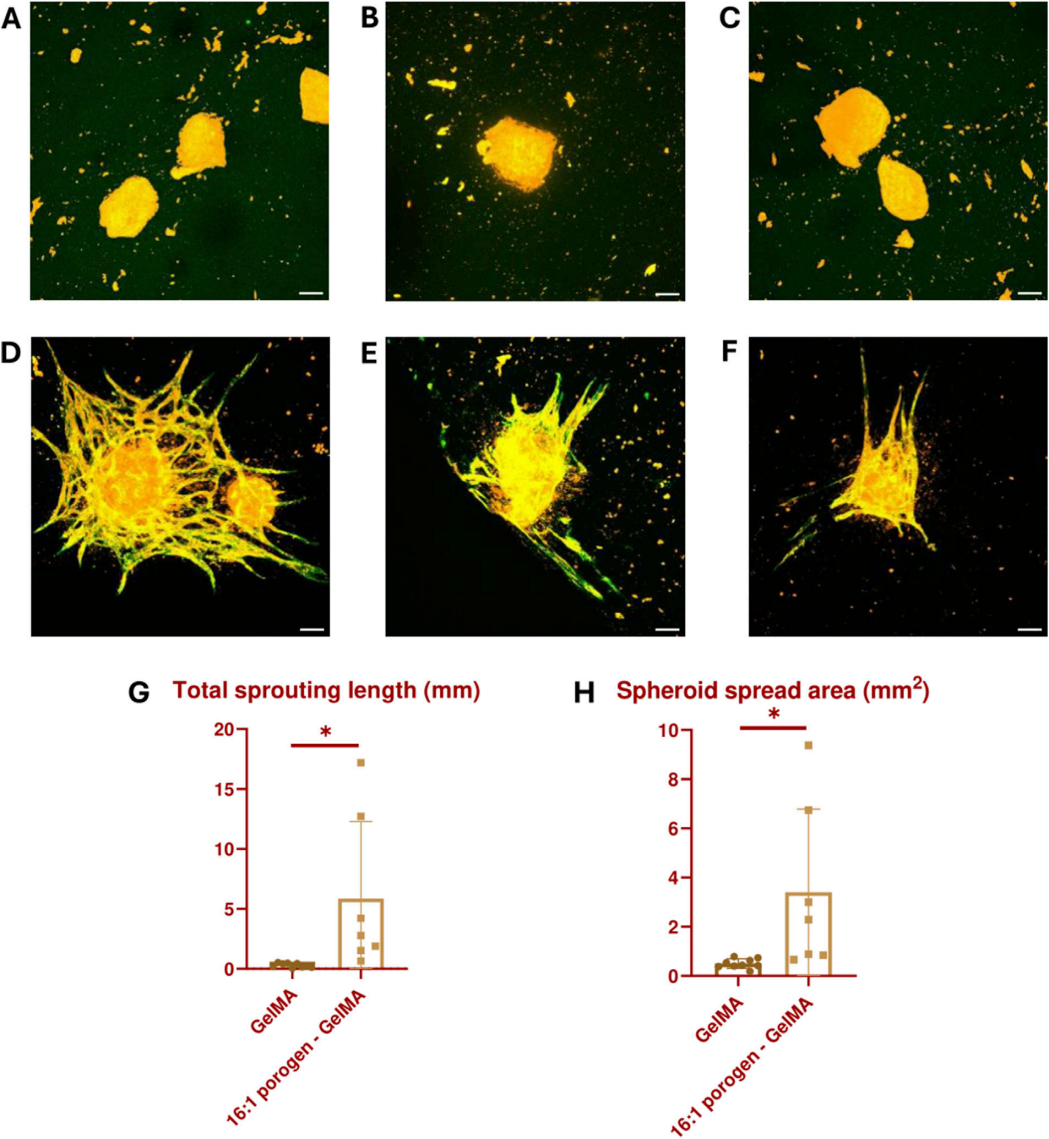
Vascularized spheroids bioprinted in GelMA **(A–C)** and 16:1 SMWA porogen-GelMA **(D–F)** after 7 days of culture. The ECs in the spheroids are stained with CellTracker Red (Orange) and CD31 antibody (Green). Scale bar = 100 µm. **(G)** Graph indicating the total sprouting length. **(H)** Graph indicating the spheroid spread area. N = 9 for GelMA and 7 for porogen-GelMA. * = *p* < 0.05.

Another assay to assess vascular development abilities is an angiogenesis assay, during which the sprouting of new blood vessels from an existing blood vessel within the biomaterial is analyzed. In this experiment, the existing blood vessels are simulated by vascularized spheroids consisting of a HUVEC and ASC coculture. These spheroids were embedded in GelMA or 16:1 porogen-GelMA and 3D bioprinted in the same shape as described in Figures 5C, D. On day 7, the printed constructs were fixed, stained and analyzed. Figures 9A–C and Figures 9D–F show images of the spheroids encapsulated in GelMA and 16:1 porogen-GelMA, respectively. The ECs in the spheroids are stained with CellTracker Red and a CD31 antibody. From these images, it is clear that the use of porogen enhanced the ability of the spheroids to form capillary sprouts. Figures 9G, H show the quantification of the sprouting angiogenesis. The spheroids encapsulated in porogen-GelMA had a total sprouting length of 5.90 mm ± 6.44 mm and spread area of 3.40 mm^2^ ± 3.38 mm^2^, which was significantly higher than the total sprouting length of 0.29 mm ± 0.18 mm and spread area of 0.49 mm^2^ ± 0.19 mm^2^ in the GelMA group. There was however a large standard deviation in the porogen-GelMA group. This could be explained by the stochastic distribution of cells and porogens inside the hydrogel when mixing them before bioprinting. Some spheroids can be closer positioned to porogen-made pores than others, better facilitating the sprouting angiogenesis. Moreover, some spheroids were able to fuse in the porogen-GelMA group, while others were not, which contributed to the formation of outliers in the data.

**FIGURE 10:**
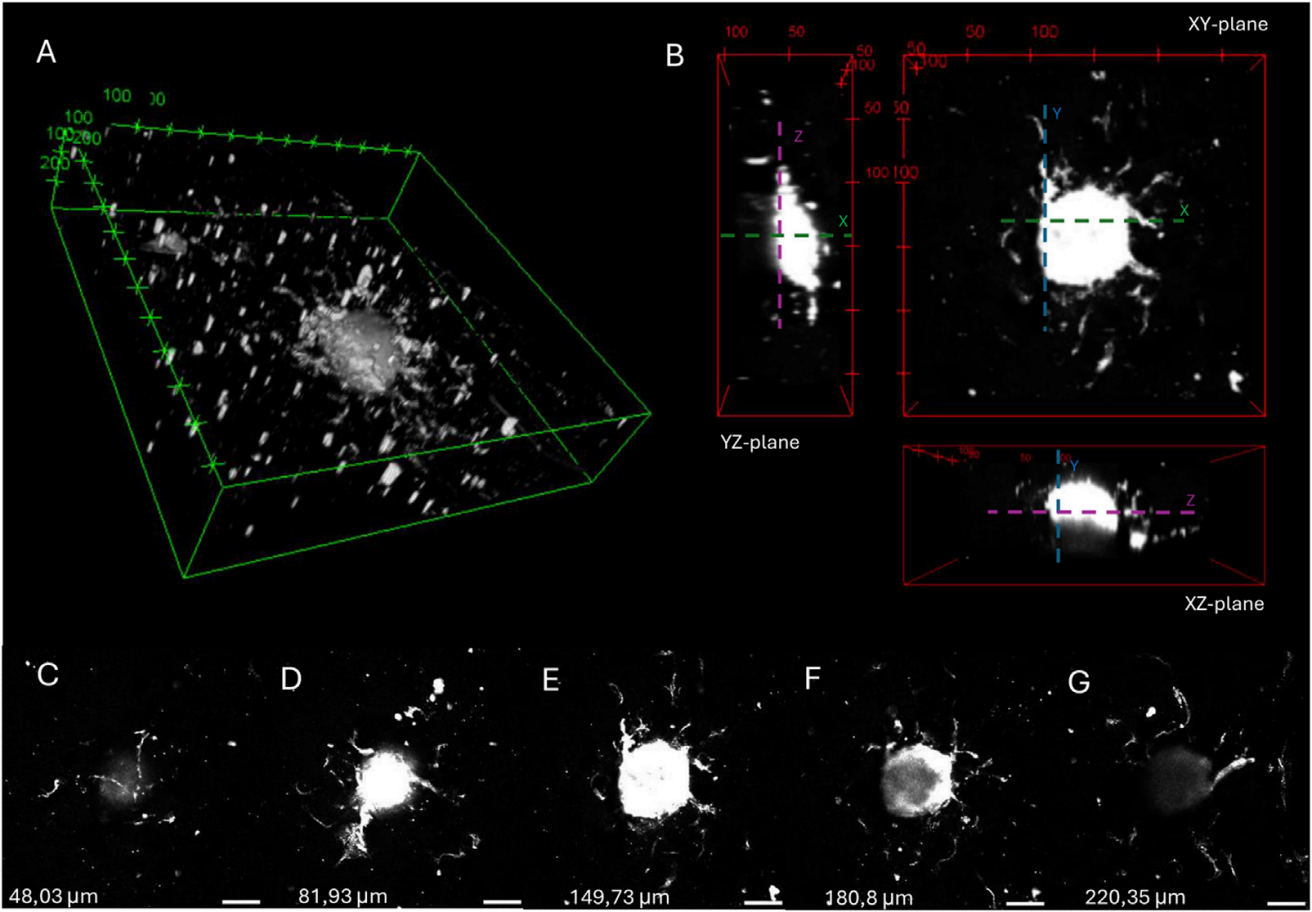
3D image **(A)** and orthogonal projections **(B)** of a spheroid embedded in 16:1 SMWA porogen-GelMA after 7 days of culture, generated by 3D viewer on ImageJ. The distance between tics is 100 and 50 µm for **(A)** and **(B)**, respectively. **(C–G)** Images of different Z-stack slices of the spheroid pictured in **(A,B)**. The *Z*-axis heights of the individual slices are mentioned in the figure. Scale bar = 100 µm.

A 3D image (see Figure 10A) and orthogonal projection (see Figure 10B) was made from one of the spheroids imaged in the angiogenesis assay described above. Figures 10C–G illustrates different z-stack slices of the hydrogel scaffold, showcasing that the capillary sprouts are able to grow in every layer of the hydrogel, surrounding the spheroid. This enables the spheroids to merge and allows sprouting angiogenesis towards an oxygen- and nutrient-rich environment.

This article presents an SMWA porogen, whose fabrication method is relatively easy to perform. The incorporation of this porogen in GelMA hydrogels retains its bioprintability and enhances viability and growth factor signaling. Moreover, it allows for vascular development in 3D bioprinted structures, paving the way for the bottom-up biofabrication of vascularized tissues.

The 3D bioprintability of the porogen-bioink combination allows for the three-dimensional localization and confinement of porosity within a multimaterial hydrogel structure, which is an advantage compared to the previously reported embedded bioprinting method in microporogen-structured collagen matrices (Reynolds et al., 2023). In the latter approach, the porosity completely encompasses the printed structure and is not confined by a 3D bioprinting method. Additionally, porogens such as Pluronic F-127 induce nanosized pores in hydrogel structures (Müller et al., 2015). By contrast, the SMWA porogen creates pores on a larger microscale, which is necessary to facilitate the assembly of microscale multicellular structures (Lu et al., 2022). Yi et al. reported similar micro-sized pores created by a phase-separation phenomenon in a GelMA and PVA combination, which is also bioprintable. However, they did not report to what extend the PVA is able to leach from the crosslinked hydrogel (Yi et al., 2022).

Unlike most reported porogens, calcium-crosslinked alginate can be dissolved and removed from the hydrogel on demand by immersing it in SLS. Alginate can be modified to be cell-interactive, and by combining 3D bioprinting with its on-demand dissolution, it allows for temporal and spatial control over these cell interactions, which can be advantageous for certain applications.

The main limitation of the SMWA porogen system is that it must be dissolved in a sodium citrate and PBS solution, which can be harmful for the cells if the incubation time is too long. Therefore, to prevent cell toxicity, it is advisable to allow for incomplete leaching of the porogen. If additional porosity is required, a higher porogen-GelMA ratio and multiple 20 min incubations in SLS with 24-h intervals can be attempted. The heterogeneous porogen size distribution has proven functional for vascular development; however, for different applications, a specific porogen size may be needed. In that case, a sieving method could be optimized to retain alginate gel porogens within a specific size range.

## 4 Conclusion

In this article, an easily manufactured SMWA porogen is presented for use as a supplement for bioinks during extrusion bioprinting. The commonly used bioink GelMA is employed as a scaffold to establish a proof-of-concept for this porogen system. The porogen-GelMA blend is 3D bioprintable, and the porogen can leach from the hydrogel during incubation in SLS. To minimize cytotoxicity, the incubation in SLS is limited to 20 min, resulting in incomplete leaching of the porogen from the hydrogel. However, a sufficient amount of pores is created during this process to enhance viability, growth factor signaling, vasculogenesis, and angiogenesis. The conducted vasculogenesis and angiogenesis assays confirmed that the fabricated vasculature developed into a 3D vascular network. This article aims to serve as a starting point for the use of SMWA porogens in 3D bioprinting. Moreover, larger bioprinted constructs with high cell densities can benefit from SMWA porogen incorporation to improve viability and facilitate faster vascularization through vascular self-assembly *in vitro* or by sprouting angiogenesis from a host organism after *in vivo* implantation.

## Data Availability

The original contributions presented in the study are included in the article/Supplementary Material, further inquiries can be directed to the corresponding author.
