# Supplementary material for: Small molecular weight alginate gel porogen for the 3D bioprinting of microvasculature
Source: Front Bioeng Biotechnol. 2024 Sep 24;12:1452477. doi: 10.3389/fbioe.2024.1452477 (PMC11458444; doi:10.3389/fbioe.2024.1452477)
Supplement: Supplementary file 2 [file DataSheet1.docx]

Supplementary Material

Small Molecular Weight Alginate Gel Porogen for the 3D Bioprinting of Microvasculature

Florian Vanlauwe^*^, Charlotte Dermaux, Sabina Shamieva, Stef Vermeiren, Sandra Van Vlierberghe, Phillip Blondeel

*** Correspondence:**

Florian Vanlauwe
Florian.Vanlauwe@UGent.be

# Supplementary Data

## Screening design

An experiment was conducted to analyze whether phosphate buffered saline (PBS) (Gibco™, 14190), sodium citrate (Sigma-Aldrich, W302600) and ethylenediaminetetraacetic acid tetrasodium salt dihydrate (EDTA) (Sigma-Aldrich, E6511) show sufficient calcium chelating abilities to dissolve low viscosity alginate (original alginate) (Sigma-Aldrich, A1112), crosslinked with CaCl_2_ (Sigma-Aldrich, C5670), in under 20 min. Sodium citrate and EDTA were dissolved at a concentration of 55 mM and 4.8 mM, respectively. Additionally, the exposure to 5000 units/l alginate lyase (Sigma-Aldrich, A1603), Dulbecco’s Modified Eagle Medium (DMEM) (Gibco™, 31966021) and the effect of the concentration of CaCl_2_ used to crosslink the alginate (13, 6.5 or 4.6 mg/ml) were also included in the analysis.

To efficiently analyze all factors in one experiment, a screening design was generated with design of experiments JMP software (Version 16). In this test, only the main effect of the factors was analyzed and the number of replicates was set to “3”. The screening design was generated following the D-optimality criterium to assure the narrowest confidence interval estimates of factor estimates. The D-efficiency of the generated design was 98,75.

The dissolution percentage for each generated factor combination was calculated as follows: a calcium-crosslinked gel of approximately 600 mg was weighed and freeze-dried immediately to determine the dry mass ($\boldsymbol{M}_{\boldsymbol{d}\boldsymbol{0}}$). Next, the dried alginate gel was incubated in the software generated factor combination for 20 min. Afterwards, the supernatant was removed and the gel was washed and incubated for one hour in double distilled water (Milli-Q). This was followed by freeze-drying to determine the second dry mass ($\boldsymbol{M}_{\boldsymbol{de}}$). The dissolution fraction was calculated from both masses using the following formula:

$$\boldsymbol{Dissolution}\left( \boldsymbol{\%} \right)\boldsymbol{=100-}\left( \frac{\boldsymbol{M}_{\boldsymbol{De}}}{\boldsymbol{M}_{\boldsymbol{D}\boldsymbol{0}}} \right)\boldsymbol{*100}$$

The standard least squares personality of the fit model platform was used to analyze the designed experiment. In Supplementary Figure 2A, the “effect summary” shows that only PBS and sodium citrate have a statistically significant effect on the dissolution of the calcium-alginate. In Supplementary Figure 2B the “prediction profiler” shows the estimated effect of the different factors on the calcium-alginate dissolution. Only the combination of sodium citrate and PBS is able to dissolve the calcium-alginate completely in 20 minutes.

## GPC analysis

In order to gain more insight in the effect of the 3 days heating at 80°C on the molecular weight of alginate, GPC analysis was performed. A Waters 610 fluid unit and a Waters 600 control unit equipped with a Waters 410 RI detector was used for the GPC analysis. Around 10 mg of the original and heated alginate were dissolved, filtered through a 0.45 µm membrane and injected in the GPC columns. A calibration curve with R² = 0.98 was obtained using dextran standards (Molecular weights (daltons): 6100, 11800, 49400, 107000, 337000, 642000).

Chromatograms for both the original and the heated alginate are depicted in Supplementary Figure 4. Supplementary Table 1 shows the weight average molecular weight (Mw), the number average molecular weight (Mn) and the polydispersity (Đ). Mw and Mn are reported to be lower for the heated alginate compared to the purchased alginate source, indicating that the molecular weight or hydrodynamic volume of the heated alginate is smaller than that of the original alginate. The Đ is also reported lower for the heated alginate, signifying that the molecular weight distribution of the heated alginate is narrower and thus contains a smaller variety of chain lengths.

# Supplementary Figures and Tables

## Supplementary Figures


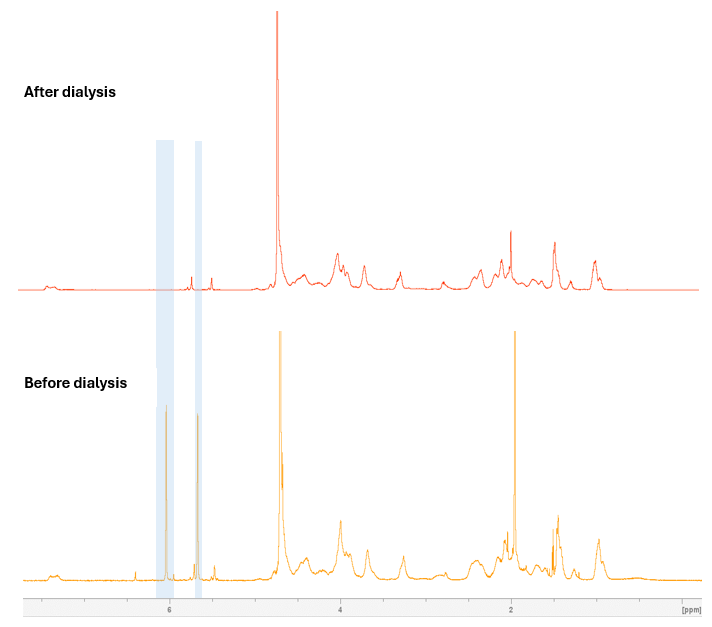


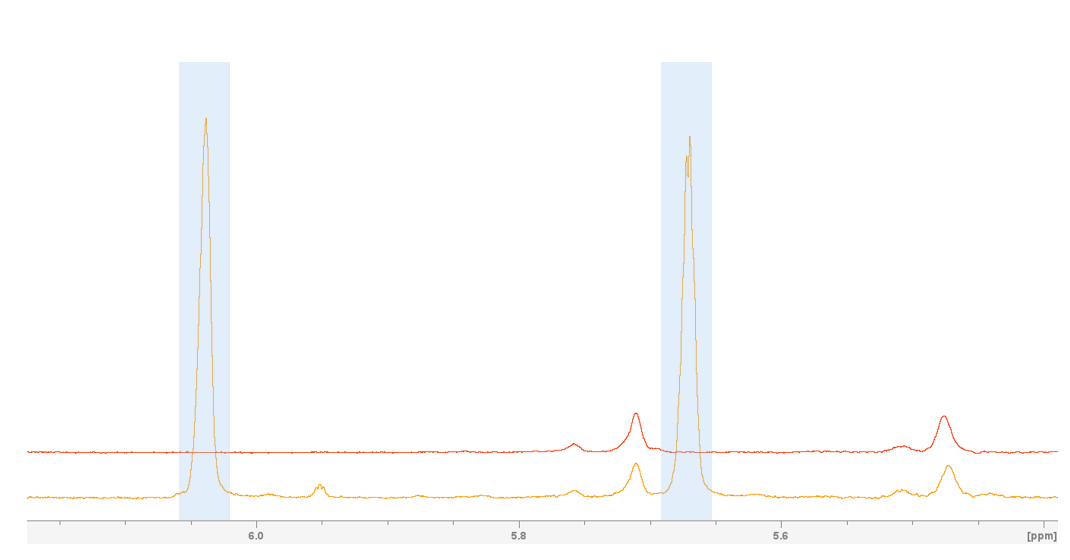


**B**

**A**

**Supplementary Figure 1.** ^1^H-NMR spectra of the synthesized GelMA, before and after dialysis: **(A)** General overview of the GelMA ^1^H-NMR spectrum. **(B)** Close-up on the proton peaks between 6.2 and 5.4 ppm. ^1^H-NMR spectroscopy was performed with the Bruker 500MHz Avance III HD at 40 °C. All samples were redissolved in D_2_O after freeze-drying the GelMA. The vinyl hydrogen peaks of the methacrylic acid, highlighted in blue, are shifted compared to the vinyl hydrogen peaks of the methacrylamides in GelMA and are absent in the dialyzed GelMA spectrum. This indicates that a single day of dialysis was effective in removing most of the residual methacrylic acid.


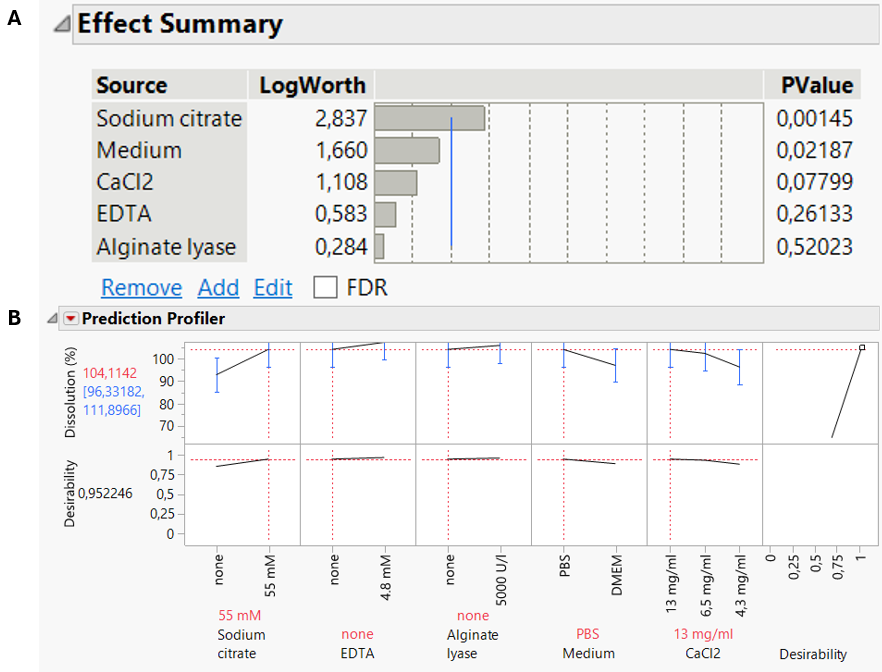


**Supplementary Figure 2.** Effect summary **(A)** and prediction profiler **(B)** generated by JMP software to screen the effect of different conditions on the dissolution of calcium-crosslinked alginate.


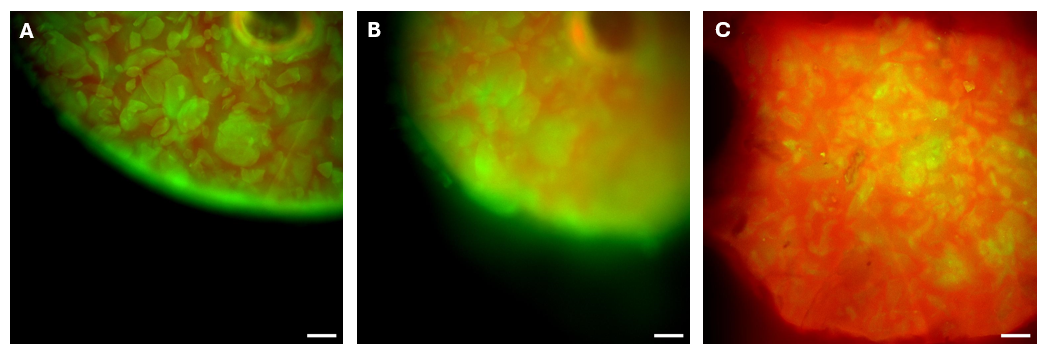


**Supplementary Figure 3.** GFP- and TRITC-filtered composite images of a 16:1 fluorescein-labelled original alginate porogen and rhodamine-GelMA blend: **(A)** Before the addition of standard leaching solution (SLS). **(B)** After 20 min of incubation in SLS. **(C)** After 2 days of incubation in SLS. Even after 2 days, a large part of the alginate is still present in the hydrogel. Scale bars = 250 µm.


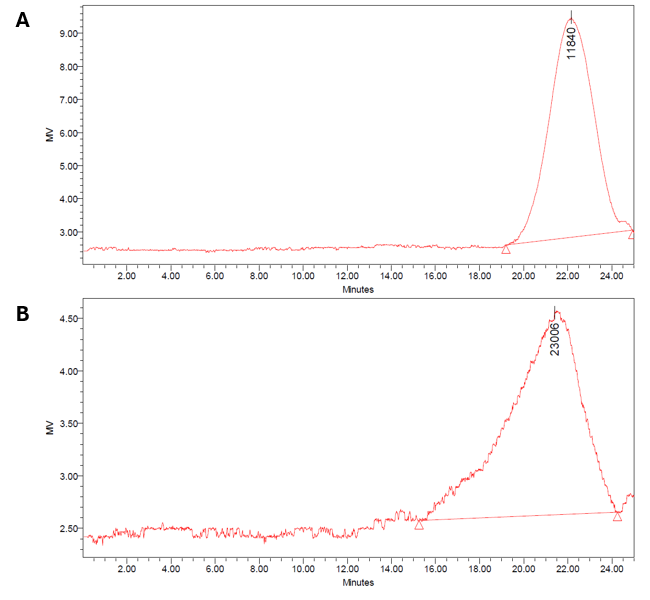


**Supplementary Figure 4.** GPC chromatogram of the heated **(A)** and the original alginate **(B)**. The chromatograms suggest a 19-24 minute retention time for the heated alginate and a 14-25 minute retention time for the original alginate. This indicates that the heated alginate has a smaller average molecular weight and is less polydisperse than the original alginate.

**
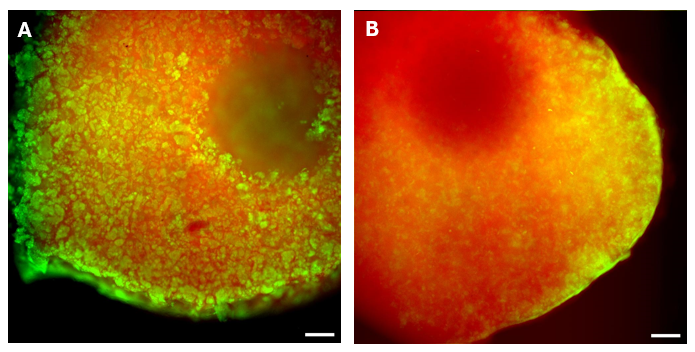
**

**Supplementary Figure 5.** GFP- and TRITC-filtered composite images of a 16:1 fluorescein-labelled SMWA porogen and rhodamine-GelMA blend: **(A)** Before the addition of SLS. **(B)** 4 days after incubation in SLS for only 20 min. After the initial treatment with SLS, the sample was incubated at 37°C in CO_2_ independent medium (Gibco, 18045-088) with a pH between 7.35-7.45, supplemented with 0.3 w/v% sodium azide (Sigma-Aldrich, S2002) until microscopy. Scale bars = 250 µm.


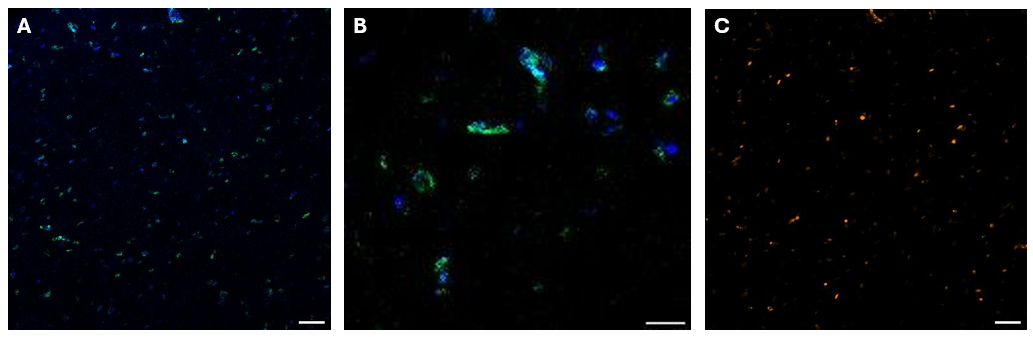


**Supplementary Figure 6.** ASC and HUVEC coculture embedded in GelMA for vascular network creation through vasculogenesis. Nuclei were stained with Hoechst and the HUVECs were stained with CellTracker Red and a fluorescein isothiocyanate conjugated CD31 antibody: **(A-B)** Hoechst and CD31 stained cells at the deepest slice of a 108 slices Z-stack, which correlates with a depth of 305.1 µm in the hydrogel. As seen in **(A)** and in the zoom image **(B)**, indicated by the dashed square in **(A)**, the CD31 antibody was able to reach the Hoechst stained cells, which signifies that the antibody was able to diffuse to the deepest region that was captured by the microscope. Scale bar = 100 µm and 50 µm for **(A)** and **(B)**, respectively. **(C)** CellTracker Red stained HUVECs on the same image Z-stack and depth as in **(A-B).** As no distinct relevant vascular structures were detected by the CellTracker Red alone, the assumption can be made that the CD31 staining of the vascular structures in the GelMA group is complete and without bias. Scale bar = 100 µm.

## Supplementary Tables

|  | **Original Alginate** | **SMWA** |
| --- | --- | --- |
| **Mw (g/mol)** | 166700 | 16190 |
| **Mn (g/mol)** | 19400 | 8620 |
| Đ | 9 | 2 |

**Supplementary Table 1.** Quantitative molar mass analysis of the original alginate and SMWA using dextran standards.
